# Prospective Observational Study of the Role of Unilateral Prophylactic Central Compartmental Lymph Node Dissection in Early Papillary Thyroid Cancer

**DOI:** 10.7759/cureus.102284

**Published:** 2026-01-25

**Authors:** Surekha Talasila, M Ramya, T Sravanthi

**Affiliations:** 1 Department of Surgical Oncology, Employees' State Insurance Corporation (ESIC) Medical College, Hyderabad, IND; 2 Department of Surgical Oncology, Gandhi Medical College, Secunderabad, IND

**Keywords:** central compartmental lymph node dissection, hypocalcaemia, papillary thyroid carcinoma, prophylactic lymph node dissection, unilateral pcnd

## Abstract

Background: In clinically node-negative (cN0) early papillary thyroid carcinoma (PTC), prophylactic central compartment lymph node dissection (PCND) remains debated because occult nodal upstaging may not translate into improved recurrence outcomes and may increase hypocalcaemia and recurrent laryngeal nerve injury. Unilateral dissection may provide a risk-limiting compromise.

Methods: This prospective single-centre study included 50 consecutive adults with T1 or T2 cN0 PTC who underwent total thyroidectomy with unilateral central compartment lymph node dissection on the tumour side. The primary outcome was occult level VI nodal metastasis and laterality pattern. Secondary outcomes included perioperative complications, particularly hypocalcaemia or hypoparathyroidism, and recurrent laryngeal nerve dysfunction.

Results: Occult central nodal metastasis was detected in 40.0% (20 of 50; 95% CI 27.6 to 53.8), resulting in pN1a upstaging. Among node-positive cases, 90.0% (18 of 20) were ipsilateral only, and 10.0% (two of 20) showed bilateral involvement in labelled tissue, with no contralateral-only disease observed. On univariable analysis, nodal positivity was associated with tumour size greater than 2 cm, capsular invasion, and multifocality. Transient hypocalcaemia occurred in 38.0% and permanent hypocalcaemia in 2.0. % Transient recurrent laryngeal nerve dysfunction occurred in 10.0% and permanent dysfunction in 2.0%. The mean postoperative stay was 3.6 days. No locoregional recurrence was detected during short-term surveillance of about 12 months.

Conclusion: In this single-centre cohort, unilateral PCND identified occult level VI disease in a substantial proportion of selected early cN0 PTC, with low permanent morbidity, but laterality interpretation and oncologic inference are limited by specimen labelling constraints and short follow-up, so the findings are hypothesis-generating and require validation in larger comparative studies.

## Introduction

Papillary thyroid carcinoma (PTC) accounts for the majority of differentiated thyroid malignancies and, in its early stages, is typically associated with excellent disease-specific survival. The persistent surgical controversy, therefore, is less about mortality and more about optimising locoregional control, minimising reoperation in the central neck, and avoiding preventable morbidity. The central compartment (level VI) is a frequent site of occult metastasis even when the neck is clinically node-negative (cN0), creating a tension between the theoretical oncologic value of prophylactic central compartment lymph node dissection (PCND) and the well-recognised risks of parathyroid and recurrent laryngeal nerve injury.

Multiple contemporary studies confirm that microscopic central nodal metastases are common, yet their clinical significance is variable. In cN0 cohorts, prophylactic dissection frequently identifies occult pN1a disease and triggers stage or risk migration, potentially influencing decisions regarding radioactive iodine and intensity of follow-up [1,2]. However, the translation of improved pathologic staging into superior long-term outcomes remains uncertain. Randomised data in small, noninvasive cN0 PTC demonstrate higher nodal upstaging with PCND but no measurable advantage in short- to mid-term structural recurrence or ablation success [3]. Similarly, a long-term randomised trial reported no meaningful differences in biochemical outcomes, reoperation, or major complications between total thyroidectomy alone and total thyroidectomy plus PCND, while suggesting increased parathyroid vulnerability through higher inadvertent parathyroid excision [4]. In parallel, large retrospective series of early disease reports overall recurrence rates that are low regardless of PCND, though omission may shift recurrence toward the central compartment, where salvage surgery is technically demanding [5].

Evidence syntheses also reflect this equipoise. Systematic review data suggest modest or inconsistent reductions in locoregional recurrence with PCND, accompanied by higher rates of hypoparathyroidism in several datasets [1]. Meta-analytic findings from randomised trials do not show a clear reduction in structural locoregional recurrence, and estimates are limited by small sample sizes and heterogeneous definitions of dissection extent and endpoints [6]. Conversely, broader pooled analyses incorporating nonrandomised studies have reported lower local recurrence with PCND but at the cost of increased hypocalcemic morbidity, underscoring the dependence of “benefit” on patient selection, surgeon expertise, and risk tolerance [7]. In older patients, routine bilateral PCND appears particularly difficult to justify because recurrence outcomes are similar while complications rise, especially beyond 75 years [8]. Across this literature, apparently discordant results often reflect meaningful heterogeneity in study design and definitions rather than true biological contradiction. Randomised trials commonly enrol lower-risk cN0 disease and use short- to mid-term recurrence or ablation endpoints, while retrospective series and pooled analyses frequently mix broader risk spectra and variable follow-up durations. In addition, central compartment dissection is not uniform across studies, varying by laterality, anatomic boundaries, inclusion of prelaryngeal or pretracheal tissue, specimen labelling practices, and definitions of transient versus permanent hypoparathyroidism. These differences influence measured nodal yield, complication rates, and the apparent magnitude of any recurrence benefit.

From an anatomic perspective, unilateral central compartment dissection targets the tumour-side paratracheal basin that is most often implicated in early spread patterns, while contralateral central metastasis appears non-universal and may be enriched in specific risk contexts. Studies examining compartment patterns and predictors of contralateral involvement suggest that nodal burden and sentinel subcompartment signals can help identify patients more likely to harbour bilateral disease, supporting a selective contralateral strategy rather than routine bilateral clearance [9-11]. In this framing, unilateral PCND is not only a pragmatic compromise but also a targeted approach that prioritises staging of the most likely compartment while aiming to limit avoidable parathyroid-related morbidity.

Within this landscape, unilateral PCND has emerged as a potential compromise for early, unilateral PTC: it may enhance staging and reduce central neck relapse while limiting the incremental morbidity associated with bilateral dissection. Long-term comparative data suggest ipsilateral dissection can preserve oncologic outcomes while markedly reducing permanent hypoparathyroidism relative to bilateral dissection [11]. Anatomical pattern studies further indicate that contralateral central metastasis is not universal and may be predicted by nodal burden and sentinel subcompartment involvement, supporting a selective rather than routine bilateral approach [10-12].

Against this background, the present prospective observational study evaluates unilateral PCND in early T1 or T2 cN0 PTC, emphasising occult nodal yield, laterality pattern based on labelled tissue where feasible, and perioperative morbidity, to clarify the staging value and safety profile of a unilateral strategy in a defined early-stage population.

## Materials and methods

Study design, setting, and duration

A prospective, single-centre observational study was conducted in the Department of Surgical Oncology of Omega Hospital, Hyderabad, Telangana, India, from March 2023 to August 2024. The study aimed to estimate occult central compartment nodal positivity, describe laterality patterns when specimen labelling allowed, and document perioperative safety outcomes after unilateral PCND in early cN0 PTC. Institutional ethics approval was obtained prior to enrolment (OH/QAD/LH-A-03, 23 February 2023).

Study population, sample size, and recruitment

Consecutive eligible patients presenting during the study period were screened and enrolled. The sample size was calculated to estimate the primary outcome with adequate precision. Assuming an anticipated occult nodal positivity proportion of 0.50, with 95% confidence and absolute precision of 0.14, the minimum required sample was approximately 49; therefore, 50 consecutive patients were recruited. The minimum required sample size was as follows: 

$n = \frac{Z^{2} \times p \times (1 - p)}{d^{2}} = \frac{1.96^{2} \times 0.50 \times 0.50}{0.14^{2}} \approx 49$

Eligibility criteria

Inclusion Criteria

Cytology confirmed PTC on preoperative fine needle aspiration cytology (FNAC) (Bethesda VI) or cytology suspicious for malignancy (Bethesda V) with final histopathology confirming PTC, T1 or T2 disease with cN0 status on clinical and radiologic assessment, fitness for surgery, and informed consent. Exclusion criteria were recurrent or metastatic disease, a T3 or T4 primary tumour, clinically or radiologically positive central or lateral lymph nodes, age below 18 years, unfitness for surgery, or refusal of consent.

Preoperative assessment and cN0 definition

All patients underwent standardised clinical evaluation and preoperative radiologic nodal assessment as per institutional practice to classify the neck as cN0. A cN0 neck was defined as the absence of palpable lymphadenopathy and the absence of radiologic features suspicious for nodal metastasis on preoperative assessment. Lymph nodes were considered suspicious when imaging demonstrated features consistent with metastatic involvement, such as abnormal nodal morphology, loss of normal architecture, or malignant-appearing internal characteristics. Patients with clinically or radiologically suspicious central or lateral nodes were excluded.

Surgical procedure

All operations were performed by the institutional surgical oncology team using standard thyroid surgery principles with meticulous identification and preservation of the recurrent laryngeal nerve and parathyroid glands. Total thyroidectomy was performed in all enrolled patients, with concurrent unilateral PCND.

Definition of dominant tumour and operative side selection

The dominant tumour was defined as the lesion judged most clinically relevant for operative planning, typically the largest focus on preoperative assessment and confirmed on final histopathology. In multifocal disease, the side containing the dominant focus guided the unilateral central compartment dissection. For isthmic or near-midline lesions, the dissection side was assigned based on the side of greater tumour bulk or extension identified preoperatively and intraoperatively, while maintaining the intent of a unilateral approach.

Anatomical extent of unilateral PCND

Unilateral central compartment dissection was performed on the tumour side within the central neck boundaries, targeting the ipsilateral paratracheal nodal basin and adjacent central fibrofatty lymphatic tissue. The intended dissection included ipsilateral paratracheal tissue along the recurrent laryngeal nerve course and the ipsilateral pretracheal and prelaryngeal tissue encountered within the operative field up to the midline. Routine contralateral paratracheal dissection was not performed. Midline tissue was managed according to intraoperative anatomy and safety considerations, while preserving the unilateral intent and avoiding deliberate contralateral clearance. Intraoperative nerve monitoring was not mandated by protocol. Recurrent laryngeal nerve preservation relied on direct identification and a meticulous dissection technique.

Specimen handling and histopathology

Thyroidectomy specimens and central compartment nodal tissue were submitted for histopathology. Specimen labelling by side and by midline component was attempted whenever feasible. When tissue separation or labelling was not possible due to en bloc removal or anatomic constraints, laterality interpretation was considered limited for that specimen. Histopathology reported tumour size, focality, laterality, lymphovascular invasion, capsular invasion, margin status, tumour variant, and extrathyroidal extension when described, including whether extension was microscopic or gross when such distinction was available. Lymph node reporting included the number of nodes retrieved, the number positive, and extranodal extension when identified.

Study outcomes

The primary outcome was occult central compartment lymph node metastasis detected on histopathology following unilateral prophylactic dissection and laterality pattern when side-specific labelling permitted classification. Secondary outcomes included perioperative complications, particularly hypocalcaemia or hypoparathyroidism and recurrent laryngeal nerve dysfunction, as well as haematoma, seroma, surgical site infection, chyle leak if encountered, and length of stay.

Definitions and ascertainment of postoperative complications

Hypocalcaemia and hypoparathyroidism were assessed clinically and biochemically according to institutional postoperative monitoring protocols. Biochemical hypocalcaemia was defined using the institutional laboratory reference range for serum calcium and/or the presence of symptoms requiring calcium supplementation. Transient dysfunction was defined as resolution within six months, and permanent dysfunction as persistence beyond six months. Recurrent laryngeal nerve dysfunction was assessed clinically. Laryngeal examination was performed when clinically indicated, such as for voice change, aspiration symptoms, or persistent concern for vocal cord dysfunction. Transient recurrent laryngeal nerve dysfunction was defined as recovery within six months, and permanent dysfunction as persistence beyond six months.

Follow-up and postoperative management

Postoperative follow-up was conducted according to institutional thyroid cancer pathways. Follow-up visits focused on complication surveillance, recovery, and thyroid cancer monitoring. Recurrence surveillance was based on clinical assessment and available institutional monitoring tools, with early locoregional recurrence defined as clinically or radiologically detected disease during follow-up. The follow-up period for the cohort extended to approximately 12 months for early recurrence surveillance.

Statistical analysis

Data were entered into a structured proforma and analysed using IBM SPSS Statistics for Windows (version 30.0, IBM Corp., Armonk, NY, USA). Continuous variables were summarised using mean with standard deviation or median with interquartile range, as appropriate. Categorical variables were expressed as frequencies and percentages. Two-sided 95% CIs for key proportions were calculated using the exact binomial method. Exploratory associations between clinicopathologic variables and nodal positivity were assessed using chi-square (χ²) or Fisher's exact testing with ORs and 95% CIs. Given the limited event numbers and sparse cells in several predictors, analyses were planned and reported as univariable. Multivariable modelling was not pursued because unstable estimates and inflated CIs were anticipated under data sparsity. A two-sided p-value below 0.05 was considered statistically significant.

## Results

Study cohort and baseline clinicopathologic profile

Fifty consecutive adults with early PTC (T1/T2) and a clinically and radiologically node-negative neck were included. All patients underwent total thyroidectomy with tumour-side unilateral PCND; side-specific specimen labelling was performed wherever feasible. The mean age was 45 years, and 66% were female (33/50; 95% CI 52.2%-77.6%). On final histopathology, tumour size was <1 cm in 24% (12/50), 1-2 cm in 42% (21/50), and 2-4 cm in 34% (17/50). Follicular variant was the most frequent subtype (40%; 20/50). Lymphovascular invasion was present in 26% (13/50), capsular invasion in 36% (18/50), and multifocality in 34% (17/50) (Table 1).

**Table 1:** Baseline profile and primary tumour pathology (N = 50) Abbreviations: N, number; n, frequency

Variable	Result (n (%))
Age, years (mean)	45
Age group: 10-25 years, n (%)	6 (12%)
Age group: 25-40 years, n (%)	16 (32%)
Age group: 40-55 years, n (%)	11 (22%)
Age group: 55-70 years, n (%)	14 (28%)
Age group: >70 years, n (%)	3 (6%)
Sex: Female, n (%)	33 (66%)
Sex: Male, n (%)	17 (34%)
Tumour size: <1 cm, n (%)	12 (24%)
Tumour size: 1-2 cm, n (%)	21 (42%)
Tumour size: 2-4 cm, n (%)	17 (34%)
Pathological stage: IA, n (%)	4 (8%)
Pathological stage: IB, n (%)	12 (24%)
Pathological stage: II, n (%)	14 (28%)
Pathological stage: III, n (%)	19 (38%)
Pathological stage: IVA, n (%)	1 (2%)
Histologic variant: Follicular, n (%)	20 (40%)
Histologic variant: Classic/Conventional, n (%)	18 (36%)
Histologic variant: Tall cell, n (%)	5 (10%)
Histologic variant: Hobnail, n (%)	2 (4%)
Histologic variant: Oncocytic, n (%)	2 (4%)
Histologic variant: Diffuse sclerosing, n (%)	2 (4%)
Histologic variant: Solid, n (%)	1 (2%)
Lymphovascular invasion: Present, n (%)	13 (26%)
Lymphovascular invasion: Absent, n (%)	37 (74%)
Capsular invasion: Present, n (%)	18 (36%)
Capsular invasion: Absent, n (%)	32 (64%)
Focality: Unifocal, n (%)	33 (66%)
Focality: Multifocal, n (%)	17 (34%)

Occult central compartment nodal metastasis and laterality

Occult central compartment lymph node metastasis was identified in 40% (20/50; 95% CI 27.6%-53.8%). Among node-positive cases (n = 20), 90% (18/20; 95% CI 69.9%-97.2%) had ipsilateral-only positivity, while 10% (2/20; 95% CI 2.8%-30.1%) had bilateral positivity based on labelled tissue submitted. No patient had isolated contralateral-only central compartment metastasis (Table 2).

**Table 2:** Central compartment nodal positivity and laterality (N = 50) Abbreviations: N, number; n, frequency.

Variable	Result (n (%))
Central node status: Positive, n (%)	20 (40%)
Central node status: Negative, n (%)	30 (60%)
Laterality among node-positive: Ipsilateral only, n (%)	18 (90%)
Laterality among node-positive: Bilateral, n (%)	2 (10%)
Laterality among node-positive: Contralateral only, n (%)	0 (0%)

Node-positive subgroup profile

Within node-positive patients (n = 20), nodal positivity clustered most frequently in the 55-70-year age band (45%; 9/20). Females constituted 55% (11/20). Tumours measuring 2-4 cm comprised 65% (13/20). Capsular invasion and multifocality were common in this subgroup (Table 3).

**Table 3:** Clinicopathologic characteristics of node-positive cases (n = 20). Abbreviations: n, frequency.

Variable	Result (n (%))
Age group: 10-25 years, n (%)	3 (15%)
Age group: 25-40 years, n (%)	5 (25%)
Age group: 40-55 years, n (%)	3 (15%)
Age group: 55-70 years, n (%)	9 (45%)
Age group: >70 years, n (%)	0 (0%)
Sex: Female, n (%)	11 (55%)
Sex: Male, n (%)	9 (45%)
Tumour size: <1 cm, n (%)	3 (15%)
Tumour size: 1-2 cm, n (%)	4 (20%)
Tumour size: 2-4 cm, n (%)	13 (65%)
Lymphovascular invasion: Present, n (%)	8 (40%)
Lymphovascular invasion: Absent, n (%)	12 (60%)
Capsular invasion: Present, n (%)	17 (85%)
Capsular invasion: Absent, n (%)	3 (15%)
Histologic variant: Follicular, n (%)	6 (30%)
Histologic variant: Classic/Conventional, n (%)	14 (70%)
Histologic variant: Other variants, n (%)	0 (0%)
Focality: Unifocal, n (%)	5 (25%)
Focality: Multifocal, n (%)	15 (75%)

Age group and tumour size distributions by central compartment nodal status

Age distribution did not differ significantly between node-positive and node-negative groups (χ²=6.94, df=4; p=0.139); descriptively, central compartment lymph node postive cases clustered more in the 55-70 year stratum (9/20, 45.0%) than central compartment lymph node negative cases (5/30, 16.7%), and none of the central compartment lymph node postive patients were >70 years (0/20, 0.0%) versus 3/30 (10.0%) in central compartment lymph node negative. In contrast, tumour size demonstrated a significant shift across categories (χ²=14.39, df=2; p<0.001): 2-4 cm tumours predominated in central compartment lymph node postive (13/20, 65.0%) compared with central compartment lymph node negative (4/30, 13.3%), whereas 1-2 cm tumours were more frequent in central compartment lymph node negative (17/30, 56.7%) than central compartment lymph node positive (4/20, 20.0%); microcarcinomas (<1 cm) were also relatively more common in central compartment lymph node negative thancentral compartment lymph node positive (9/30, 30.0% vs 3/20, 15.0%), indicating that increasing tumour size particularly >2 cm tracked closely with occult central nodal metastasis in this cohort (Table 4).

**Table 4:** Multi-category clinicopathologic variables versus central compartment nodal positivity Pearson chi-square tests were used to compare the overall distribution across categories. The chi-square (χ²) statistic and p-value are reported once per variable (overall row) and not repeated for sub-categories. Abbreviations: CCLN, central compartment lymph node; df, degrees of freedom; +: positive; -: negative

Variable	CCLN+ (n=20) n (%)	CCLN- (n=30) n (%)	χ² (df)	p-value
Age group (overall)	20 (100.0%)	30 (100.0%)	6.94 (4)	0.139
Age group: 10-25 years	3 (15.0%)	3 (10.0%)	-	-
Age group: 25-40 years	5 (25.0%)	11 (36.7%)	-	-
Age group: 40-55 years	3 (15.0%)	8 (26.7%)	-	-
Age group: 55-70 years	9 (45.0%)	5 (16.7%)	-	-
Age group: >70 years	0 (0.0%)	3 (10.0%)	-	-
Tumour size (overall)	20 (100.0%)	30 (100.0%)	14.39 (2)	<0.001
Tumour size: <1 cm	3 (15.0%)	9 (30.0%)	-	-
Tumour size: 1-2 cm	4 (20.0%)	17 (56.7%)	-	-
Tumour size: 2-4 cm	13 (65.0%)	4 (13.3%)	-	-

Binary predictors of central compartment nodal positivity

Binary comparisons using Fisher’s exact testing highlighted three clinicopathologic features with strong associations with central nodal positivity. Tumour size >2 cm was present in 13/20 (65.0%) of central compartment lymph node-positive patients compared with 4/30 (13.3%) of central compartment lymph node-negative patients (OR 12.07; 95% CI 2.98-48.82; p=0.000245), indicating substantially higher odds of occult central metastasis above this size threshold. Capsular invasion showed the most pronounced relationship: 17/20 (85.0%) of node-positive patients exhibited capsular invasion versus 1/30 (3.3%) of node-negative patients (OR 164.33; 95% CI 15.81-1707.80; p<0.001), consistent with a markedly enriched risk profile when capsular invasion is present. Multifocality was also strongly associated, occurring in 15/20 (75.0%) of central compartment lymph node-positive patients versus 2/30 (6.7%) of central compartment lymph node-negative patients (OR 42.00; 95% CI 7.26-243.07; p<0.001). By comparison, male sex (45.0% vs 26.7%; OR 2.25; p=0.229) and lymphovascular invasion (40.0% vs 16.7%; OR 3.33; p=0.100) did not reach statistical significance in this cohort, although both showed directional increases among node-positive patients (Table 5).

**Table 5:** Binary clinicopathologic predictors of central compartment nodal positivity Fisher’s exact tests with odds ratios were employed. Abbreviations: CCLN, central compartment lymph node; CI, confidence interval; OR, odds ratio; +: positive; -: negative

Variable	CCLN+ (n=20) n (%)	CCLN- (n=30) n (%)	OR	95% CI (lower)	95% CI (upper)	p-value
Tumour size >2 cm (Yes vs No)	13 (65.0%)	4 (13.3%)	12.07	2.98	48.82	0.000245
Sex: Male (Yes vs No)	9 (45.0%)	8 (26.7%)	2.25	0.68	7.44	0.229
Lymphovascular invasion: Present (Yes vs No)	8 (40.0%)	5 (16.7%)	3.33	0.90	12.38	0.100
Capsular invasion: Present (Yes vs No)	17 (85.0%)	1 (3.3%)	164.33	15.81	1707.80	<0.001
Multifocality: Present (Yes vs No)	15 (75.0%)	2 (6.7%)	42.00	7.26	243.07	<0.001

Univariable (unadjusted) logistic regression for predictors of central compartment nodal positivity

Univariable logistic regression yielded estimates that were directionally and numerically concordant with the contingency-table analyses, reinforcing the same predictors as statistically significant. Tumour size >2 cm remained associated with higher odds of nodal positivity (OR 12.07; 95% CI 2.98-48.82; Wald p=0.000476). Capsular invasion continued to demonstrate a very large effect size (OR 164.33; 95% CI 15.81-1707.80; Wald p<0.001), and multifocality remained a strong predictor (OR 42.00; 95% CI 7.26-243.07; Wald p<0.001). Male sex showed a non-significant association (OR 2.25; 95% CI 0.68-7.44; Wald p=0.184), while lymphovascular invasion approached but did not meet conventional significance thresholds (OR 3.33; 95% CI 0.90-12.38; Wald p=0.072). Multivariable modelling was not pursued because sparse cell counts, particularly for capsular invasion in the node-negative group, would likely yield unstable adjusted estimates and wide, unreliable CIs (Table 6).

**Table 6:** Univariable logistic regression for predictors of central nodal positivity (N = 50) Models are univariable, and predictors are binary; therefore, logistic regression ORs mirror contingency-table ORs. Regression results are provided to report modelling-based estimates. Abbreviations: CI, confidence interval; OR, odds ratio.

Predictor (reference)	OR	95% CI (lower)	95% CI (upper)	Wald p-value
Tumour size >2 cm (≤2 cm)	12.07	2.98	48.82	0.000476
Capsular invasion: Present (Absent)	164.33	15.81	1707.80	<0.001
Multifocality: Present (Unifocal)	42.00	7.26	243.07	<0.001
Sex: Male (Female)	2.25	0.68	7.44	0.184
Lymphovascular invasion: Present (Absent)	3.33	0.90	12.38	0.072

Postoperative hospital stay and perioperative complications

The mean postoperative hospital stay was 3.6 days. Postoperative events included hematoma in 2.0% (1/50; 95% CI 0.4%-10.5%) and seroma in 12.0% (6/50; 95% CI 5.6%-23.8%). Transient hypocalcaemia occurred in 38.0% (19/50; 95% CI 25.9%-51.8%), while permanent hypocalcaemia occurred in 2.0% (1/50; 95% CI 0.4%-10.5%). Recurrent laryngeal nerve dysfunction was transient in 10.0% (5/50; 95% CI 4.3%-21.4%) and permanent in 2.0% (1/50; 95% CI 0.4%-10.5%) (Table 7).

**Table 7:** Postoperative hospital stay and perioperative complications (N = 50)

Outcome	n/N or mean	Percentage (%)
Postoperative hospital stay (days)	Mean 3.6	-
Hematoma	1/50	2.0
Seroma	6/50	12.0
Transient hypocalcaemia	19/50	38.0
Permanent hypocalcaemia	1/50	2.0
Transient recurrent laryngeal nerve dysfunction	5/50	10.0
Permanent recurrent laryngeal nerve dysfunction	1/50	2.0

Follow-up

Patients were followed for up to approximately 12 months for early locoregional recurrence surveillance. No locoregional recurrence was identified (0/50; two-sided exact 95% CI: 0%-7.1%).

## Discussion

In this prospective study of clinically and radiologically node-negative PTC managed with total thyroidectomy plus unilateral PCND, the findings are best interpreted as descriptive and hypothesis-generating rather than definitive. Occult level VI metastasis was detected in 40%, supporting prior observations that microscopic central disease is common even in cN0 presentations and that prophylactic dissection can reveal pN1a disease with potential implications for risk stratification and follow-up planning [1,2,13,14]. A predominantly ipsilateral pattern was observed among node-positive cases, with bilateral positivity identified in a small minority, but laterality inferences should be interpreted cautiously because side-specific conclusions depend on the completeness of specimen separation and labelling. The exploratory association analyses suggested that larger tumour size, capsular invasion, and multifocality clustered with nodal positivity in this dataset, yet these effect estimates are based on limited event numbers and should be viewed as signals for confirmation rather than as stable predictors [2,15]. From a safety perspective, permanent morbidity was uncommon in this cohort, while transient hypocalcaemia was frequent, a pattern that underscores the need to interpret complication rates in the context of institutional monitoring intensity and definitional thresholds [1,4,7].

A few aspects of the present results may diverge in magnitude from parts of the existing literature. The 40% occult nodal positivity is lower than some datasets reporting substantially higher central metastasis detection with routine PCND in cN0 PTC (for example, the >50% range reported in certain cohorts) [1,2] and far below rates observed in selected higher-risk populations (e.g., patients with lateral node disease where microscopic central involvement is extremely common) [16]. Conversely, the observed transient hypocalcaemia rate (38%) sits toward the higher end of what is often cited, even though permanent hypocalcaemia remained low, and this pattern contrasts with some randomised syntheses that did not show statistically robust increases in biochemical complications with PCND, albeit with wide uncertainty and heterogeneous definitions [3,6]. Finally, the very low bilateral positivity signal (10% among node-positive) is smaller than contralateral central involvement rates described in studies that performed bilateral central dissection and compartment-by-compartment labelling [11], highlighting how dissection extent and specimen mapping can shape observed laterality.

Several explanations can plausibly account for these differences without implying true biological contradiction. Case-mix matters: this cohort was restricted to T1/T2, cN0 disease, whereas higher occult nodal rates are often reported in settings enriched for aggressive histology, lymphatic invasion, or different thresholds for surgical indication [2,16]. Methodology also matters. Studies that systematically perform bilateral dissection with meticulous subcompartment labelling can detect contralateral micrometastasis that a unilateral strategy will not sample, and “absence of contralateral-only disease” in a unilateral design should be interpreted as no detected isolated contralateral signal rather than proof of impossibility [11]. The transient hypocalcaemia frequency can be influenced by perioperative calcium/parathyroid hormone surveillance intensity, supplementation thresholds, and definitional stringency factors that often vary across centres and trials [4,6]. Finally, follow-up duration shapes oncologic interpretation: the absence of early locoregional recurrence over ~12 months is reassuring but cannot adjudicate the longer-horizon question that dominates the controversy of whether PCND changes structural recurrence patterns or mainly shifts where recurrence occurs [5].

The novel contribution of this study lies in providing prospective, real-world evidence focused specifically on a unilateral prophylactic strategy in early cN0 PTC within an Indian tertiary setting, with explicit reporting of (i) occult nodal yield, (ii) laterality pattern, and (iii) perioperative morbidity alongside exploratory clinicopathologic correlates. The observation that ipsilateral-only positivity dominated and contralateral-only disease was not seen is directionally consistent with the growing “selective contralateral” paradigm: contralateral central dissection may be reserved for patients with predictors of bilateral spread or sentinel-compartment involvement rather than used routinely [10-12]. In practical terms, these data support unilateral PCND as a plausible compromise approach that preserves staging benefits while aiming to limit the morbidity historically linked to more extensive bilateral clearance [8,9].

Future work should move from descriptive feasibility to decision-quality evidence. Larger multicentre prospective cohorts or pragmatic comparative designs should evaluate unilateral PCND against thyroidectomy alone and against bilateral PCND, with standardised subcompartment labelling and longer follow-up to test whether unilateral PCND meaningfully reduces central-compartment recurrence or merely refines staging [4,5]. Risk-stratified algorithms could integrate readily available predictors suggested here (e.g., tumour size >2 cm, capsular invasion, multifocality) together with intraoperative nodal burden or sentinel subcompartment cues that have shown promise for anticipating contralateral disease [10-12]. Given that parathyroid morbidity remains the critical trade-off, future protocols should also explicitly test mitigation strategies and report uniform definitions of transient/permanent dysfunction to improve cross-study comparability [6,7].

This study should be interpreted in light of limitations. The sample size (n=50) was intentionally pragmatic, leaving limited power for stable multivariable modelling and producing wide CIs for some association estimates. The single-centre observational design and lack of a contemporaneous control group (thyroidectomy alone or bilateral PCND) constrain causal inference regarding the net benefit of unilateral PCND. While laterality patterns were assessed with side-specific labelling “where feasible,” incomplete or variable specimen mapping, along with surgeon-judgement management of midline tissue, may affect precision in interpreting bilateral signals. The follow-up period (~12 months) is adequate for early safety surveillance but is too short to resolve late recurrence questions in an indolent malignancy, a limitation repeatedly emphasised even in randomised and large retrospective studies.

## Conclusions

In this single-centre prospective cohort of early clinically and radiologically node-negative PTC managed with total thyroidectomy and tumour-side unilateral PCND, occult central compartment nodal disease was frequently identified, with a predominantly ipsilateral distribution when laterality labelling was available. Perioperative morbidity was predominantly transient, with low permanent hypocalcaemia and low permanent recurrent laryngeal nerve dysfunction. Because laterality assessment depended on specimen separation and labelling where feasible, and because follow-up was limited to approximately 12 months, these findings should be interpreted as suggestive rather than confirmatory, and they do not establish long-term oncologic benefit. Unilateral PCND may be considered for carefully selected early cN0 patients in whom nodal staging is likely to influence adjuvant decision making and surveillance intensity, but larger comparative studies with standardised specimen mapping and longer follow-up are required before broader generalisation.
